# Four Cases of Cerebro-Spinal Meningitis

**Published:** 1865-02

**Authors:** P. Gervais Robinson

**Affiliations:** Surgeon P. A. C. S.


ART. II.—*Four Cases of Cerebro-Spinal Meningitis.* Reported by P. GERVAIS ROBINSON, Surgeon P. A. C. S.

An article in the August number of the "Confederate States Medical and Surgical Journal," on Cerebro-Spinal Meningitis, by Surgeon G. A. Moses, of Mobile, Ala., has induced me to make a few remarks upon the same form of disease, which showed itself in the winter of '62–'63, in the command to which I was then attached, the Twenty-Second North Carolina regiment, encamped at Moss Neck, on the Rappahannock, six or seven miles below Fredericksburg. There occurred four cases in close succession, without apparent or appreciable cause, but it was a source of congratulation that this truly formidable malady was thus limited in its ravages. All the soldiers attacked were members of the same company; three were conscripts and had been in camp but a short time, perhaps little more than a month, the fourth had served from the commencement of hostilities. Of the three conscripts two were brothers, and the third their brother-in-law. These all died of the disease, the veteran alone recovered. In the first case, the phenemena were few and very obscure, and not sufficient to lead to a just appreciation of its gravity. The patient complained on the first day of a dull constant headache, which was persistent and from which he could obtain no relief. His pulse was about eighty beats in the minute, and not perceptibly abnormal in calibre or force; his tongue presented no departure from a state of health, and the only other symptom beside the headache which indicated disease, was a constipated condition of the bowels. There was at no time delirium, and no variation in the size of the pupils. After the first twenty-four hours there was perhaps some disposition to drowsiness, from which, however, the patient was easily aroused. On the morning of the fifth day he became profoundly comatose, and died the same day. This case was obscure from its beginning to its termination; indeed, up to the third morning there was so little apparent cause of disease that I was inclined to regard the complaints of the patient with suspicion as to their honesty, and the case as one of malingering; I asked, however, the advice of a surgeon of the brigade of larger experience than myself, who concurred in my suspicions, but guided by the severe and persistent headache, I was induced to withhold such harsh judgment, and to treat the case as disease of the brain. I ordered a carthartic of oleum ricini et hyd. chl. mit., which produced no effect, when the oleum tiglii was used successfully. Cold applications to the head by means of wet cloths, with blisters to the nape of the neck and along the spine, completed the list of remedial agents, with no evident influence upon the course of the disease.

Autopsy a few hours after death revealed the true state of the case. On opening the calvaium, the "dura mater" appeared sufficiently healthy, but not so the membranes beneath. The surface of the brain was very much and generally injected, and there was a very extensive effusion between the "arachnoid" and "pia mater," with more condensed and coagulated patches, of a yellowish color here and there, and more especially along the longitudinal sinus of the cerebrum. Here and there these membranes were adherent, but could, with care, be separated with the handle of the scalpel. These deposits or exudations of lymph were found likewise about the base of the brain, and chiefly and in greatest quantity about the "medulla oblongata" and commencement of the cord. No effusion was found in the lateral ventricles, and the substance of the brain presented no appearance of having participated in the inflammation of its membranes.

This case is interesting and important as showing that there is no fixed or certain relation between the pathological condition of the diseased brain and the phenomena manifested, and that the most fatal form of cerebral affection can exist and progress to an unfavorable issue without creating more than a vague suspicion of its gravity.

On the same day that this soldier died, his brother complained of a dull and persistent headache, and in the evening became violently and suddenly delirious, raving and struggling to such a degree as to require the aid of three or four men to restrain him. In him the pulse was about ninety beats to the minute, full and strong, and the bowels closely constipated. By the use of cold water, poured over the head in considerable quantity, the more violent paroxysms were controlled, and by the steady application of wet cloths, in eighteen hours, his delirium subsided and reason was restored. The bowels were moved by the croton oil; and blisters applied to the nape of the neck and spine constituted the list of remedies used in this the first stage of the disease. From this time until about the end of the fifth day the disease appeared to be quiet and inactive, the patient being tranquil and quite rational, and partaking of such light nourishment as could be procured in camp. During this period of intermission, there were no very remarkable symptoms of disease, the pulse being almost quite normal, the tongue and skin presenting no very peculiar or characteristic conditions, and perhaps the only appreciable sign of any lesion of the brain was exhibited in the paralysis of the sensory root of the fifth pair of nerves, thus destroying the sensibility of the face. Towards the end of the fifth day the functions of the optic began to be interfered with, and vision became impaired, together with dilatation of the pupils. It was noticed, too, during this interval, that the sensibility of the "portio mollis" was much increased, the hearing unnaturally acute, and the patient's attention would be attracted by the slightest noise, and he would even remark upon what was said in a low tone at some distance from his bed. A part of this time he could not recognize a watch held at his nose nor feel a severe pinch of his cheek. From this he became much prostrated and sank into a state of collapse, and soon, profound coma supervening, in this condition he continued until about the seventh day of disease, when death, apparently dependent upon paralysis of the respiratory nerves, closed the scene.

The third case (this soldier being a brother by marriage of the two first) followed close upon the second and was ushered in with the like symptoms, firstly complaining of severe headache, succeeded by violent and maniacal delirium, requiring physical force to restrain his muscular efforts. Its course was very similar to that of the preceding throughout, except a variation in the paralysis apparent. In this the "portio mollis" was affected inversely, and the patient quite deaf after the third day. The delirium subsided as readily, and there was the same deceptive intermission in the march of the disease. In this case, however, this intermediate stage was more protracted, and that of collapse followed by coma came on about the sixth, and death about the ninth day of disease. The variation in the treatment of this case was in the administration of small doses of hyd. chl. mit., commenced as soon as practicable and continued until a gentle ptyalism was produced. In both, during the stage of intermission, the careful use of stimulants was deemed appropriate, and in the state of collapse with a liberality proportionate to the gravity of the indications. Stimulating enemata were also tried in the last stage, but with no apparent influence.

The fourth case followed before the termination of the third, and its approach was marked by symptoms of cerebral disease similar to the last, viz: violent headache, succeeded by delirium, which was, however, more tractable, and did not partake of the raving madness of the two preceding. The delirium was subdued and the patient restored to reason in the course of thirty-six hours, and although the pain in the head continued for some days, there was no untoward symptom, and he gradually improved until, about the sixth or seventh day, convalescence was fairly established.

An autopsy of the second and third cases presented the same general appearances as the first, except that in these the limphy exudations were more extensive and there was much greater injection of the vessels of the membranes. In these, too, the lateral ventricles were filled to their greatest capacity with fluid. The brain pulp in neither case presented any appearance of inflammation. The accumulation of thick yellow lymph was greatest about the base of the brain, the commissure of the optic nerves, "medulla oblongata" and commencement of cord. In the first case the thoracic and abdominal viscera were examined and found healthy. These autopsies were all made in the presence of several surgeons, in order to insure a correct appreciation of the signs of lesion which were found.

This malady, occurring as it did suddenly and without apparent cause, and being limited to four cases by no circumstances or influences that could be appreciated, exhibited a malignancy second to none in the category of epidemic diseases.

On inquiry I found that cases had occurred analogous in character in various locations of the C. S. army, and likewise among the negro population on various plantations further south. In some there had been pulmonary, and in others complications partaking rather of the nature of catarrhal, affections. In the cases here reported there had been no such complications, but during the same winter I had lost a case of what was considered "pneumonia," in which cerebral symptoms supervened after resolution of the lung had been well established, and from which the patient succumbed, exhausted by repeated paroxysms of convulsions. On reflection, it seems probable that this case was affected by the same malignant influences which caused the other cases here recorded.

I have been induced to report these cases in the hope that others who have had an experience of the same malady will not withhold their's from the profession, and thus we may possibly arrive at some data as to the conditions attending this deplorable disease, looking to a discovery of its causes and prevention, if not to its successful treatment.

Three of the cases here reported present the remarkable feature of having occurred in individuals so nearly connected by blood, being conscripts but a short time in camp, having undergone but little of the hardships of a soldier's life, living in the same hut and partaking of their food at a common mess.

It may reasonably be asked whether these circumstances had not great influence in the extension, at least, of the disease; these soldiers being united by common sympathies and common interests, which established a physical and mental parallelism favorable to the invasion of the same morbific causes, and guiding them, thus introduced, in their progress in the same direction and to the like termination.

